# Neurodegeneration and Glial Response after Acute Striatal Stroke: Histological Basis for Neuroprotective Studies

**DOI:** 10.1155/2016/3173564

**Published:** 2016-12-05

**Authors:** Rafael R. Lima, Luana N. S. Santana, Rafael M. Fernandes, Elder M. Nascimento, Ana Carolina A. Oliveira, Luanna M. P. Fernandes, Enio Mauricio N. dos Santos, Patrycy Assis N. Tavares, Ijair Rogério dos Santos, Adriano Gimarães-Santos, Walace Gomes-Leal

**Affiliations:** ^1^Institute of Biological Sciences, Laboratory of Functional and Structural Biology, Federal University of Pará, 66075-900 Belém, PA, Brazil; ^2^Institute of Biological Sciences, Laboratory of Experimental Neuroprotection and Neuroregeneration, Federal University of Pará, 66075-900 Belém, PA, Brazil

## Abstract

Stroke is a leading cause of death and neurological disability worldwide and striatal ischemic stroke is frequent in humans due to obstruction of middle cerebral artery. Several pathological events underlie damage progression and a comprehensive description of the pathological features following experimental stroke in both acute and chronic survival times is a necessary step for further functional studies. Here, we explored the patterns of microglial activation, astrocytosis, oligodendrocyte damage, myelin impairment, and Nogo-A immunoreactivity between 3 and 30 postlesion days (PLDs) after experimental striatal stroke in adult rats induced by microinjections of endothelin-1 (ET-1). The focal ischemia induced tissue loss concomitant with intense microglia activation between 3 and 14 PLDs (maximum at 7 PLDs), decreasing afterward. Astrocytosis was maximum around 7 PLDs. Oligodendrocyte damage and Nogo-A upregulation were higher at 3 PLDs. Myelin impairment was maximum between 7 and 14 PLDs. Nogo-A expression was higher in the first week in comparison to control. The results add important histopathological features of ET-1 induced stroke in subacute and chronic survival times. In addition, the establishment of the temporal evolution of these neuropathological events is an important step for future studies seeking suitable neuroprotective drugs targeting neuroinflammation and white matter damage.

## 1. Introduction

Stroke is a devastating condition and a leading cause of death and functional disability worldwide [1, 2]. This central nervous system (CNS) disorder is characterized by obstruction of blood vessels (ischemic stroke) or their rupture (hemorrhagic stroke) leading to metabolic collapse and a multitude of secondary mechanisms, including excitotoxicity, inflammation, metabolic acidosis, apoptosis, periinfarct depolarization, and oxidative stress [1, 2].

Following stroke an intense inflammatory response takes place, characterized by recruitment of neutrophils [3, 4], microglial activation [3, 5–7], and astrocytosis [8, 9]. Microglia activation plays both beneficial and detrimental actions following experimental stroke [5, 10, 11]. Inhibition of microglial activation with minocycline induces conspicuous neuroprotection following focal ischemia [12–14], but other experimental reports suggest that microglia may be also beneficial following stroke [10, 15–17].

Astrocytes are activated following both acute [18, 19] and chronic [20] neural disorders. These glial cells play both detrimental and beneficial actions following stroke and spinal cord injury (SCI) [21]. Astrocyte activation may contribute to ischemic damage in the hippocampus by activation of N-methyl-D-aspartate (NMDA) extrasynaptic receptors, a phenomenon involving calcium signaling [19]. On the other hand, astrocytes seem to mediate endogenous neuroprotection following stroke, a phenomenon involving activation of a glial-specific purinergic receptor, P2Y(1)R, and inositol 1,4,5 trisphosphate IP(3)/Ca^2++^ signaling [22].

Following stroke and SCI, neuroplasticity is inhibited by white matter- (WM-) associated proteins, including Nogo-A and proteoglycans [23]. Chondroitin sulfate proteoglycans (CSPS) are associated with astrocytosis and contribute to inhibition of axonal regeneration following stroke and SCI [24]. Nogo-A is an oligodendrocyte-associated protein and a major inhibitor of axonal sprouting [25, 26]. CSPS degradation with chondroitinase ABC enhances axonal sprouting following SCI [27, 28] and stroke [29, 30]. Similar findings have been found using a neutralizing antibody against Nogo-A [31–34].

In our previous investigations, we have described the patterns of microglial activation [3, 4, 12, 35], astrocytosis [18], and WM damage [3, 4, 35, 36] using experimental models of stroke, excitotoxicity, and SCI. Nevertheless, the pathological events were described up to 7 days from the disease onset. It has been suggested that WM damage with concomitant neuroinflammation is a long-lasting phenomenon even following human stroke [37]. In addition, it has been reported that the expression of Nogo-A is associated with inhibition of neuroplasticity following experimental stroke [31, 32].

Middle cerebral artery occlusion (MCAO) is a common pathological event in human stroke in which striatum and parietal cortex are damaged [37]. A comprehensive descriptive study on the basic neuropathology in both acute and chronic survival times following experimental striatal stroke is a fundamental step for future investigations seeking for neuroprotective and neuroregenerative therapies. In this study, we describe the patterns of microglia activation, astrocytosis, oligodendrocyte damage, Nogo-A immunoreactivity, and myelin impairment from 3 days to 30 days following focal ischemia induced by microinjections of endothelin-1 (ET-1) into the rat striatum.

## 2. Material and Methods

### 2.1. Experimental Animals

Male adult Wistar rats (290–300 g) were obtained from the Central Animal Facility in the Federal University of Pará. All animals were housed under standard conditions with food and water available* ad libitum*.

### 2.2. Ethics Statement

All procedures were approved by the Ethics Committee on Experimental Animals of the Federal University of Pará (CEPAE-UFPA), under license BIO 038-12. All experimental procedures followed the Principles of laboratory animal care (NIH Publication number 86-23, revised 1985). All efforts were made to avoid animal suffering and distress.

### 2.3. Stroke Model

Animals were deeply anesthetized with an intraperitoneal injection of a mixture of ketamine hydrochloride (90 mg/kg, i.p.) and xylazine hydrochloride (10 mg/kg, i.p.) and positioned in a stereotaxic apparatus after abolishment of their corneal reflex. After craniotomy, 80 pMol of ET-1 (Sigma-Aldrich, USA) in 1 *μ*L of sterile saline was injected into the rat striatum (*N* = 5 per survival time) over a period of 2 min using a glass capillary micropipette. The pipette was left in place for 3 min before being slowly withdrawn. Control animals were injected with the same volume of sterile saline (*N* = 12). We used the following stereotaxic coordinates for the injection (in millimeters relative to bregma): 2.5 mm lateral; 1.2 mm posterior; and 4.5 mm below from the pial surface. After surgery, animals were allowed to rest in their cages (maximum 4 animals per cage) with water and food* ad libitum* until postlesion days ( PLDs) 3, 7, 14, and 30. This stroke model was established in our previous reports [3, 13]. The mortality rate is less than 5% in this stroke model.

### 2.4. Perfusion and Histological Procedures

Following the described survival times, animals were deeply anesthetized with ketamine hydrochloride (90 mg/kg, i.p.) and xylazine hydrochloride (10 mg/kg, i.p.) and transcardially perfused with heparinized 0.9% phosphate-buffered saline (PBS) followed by 4% paraformaldehyde in 0.2 M phosphate buffer. Surgical manipulation was performed only after both corneal and the paw withdraw reflexes were abolished. Brains were removed from the skull, post-fixed for 24 h in the same fixative, and cryoprotected in increasing concentrations of sucrose–glycerol solutions over 7 days. The brains were then frozen in TissueTek and sectioned in order to obtain 20 and 50 *μ*m coronal sections using a cryostat (Carl Zeiss/Micron, Germany). Sections were mounted onto gelatinized slides and air-dried for 24 h. Slides were stored in a freezer at −20°C for posterior histopathological analysis.

### 2.5. Gross Histopathology and Immunohistochemistry

The ischemic lesion area was visualized in coronal sections (50 *μ*m thick) stained with cresyl violet. The site of ET-1 injection was recognized by tissue pallor associated with loss of cell bodies as described in our previous studies [3, 13, 38].

To analyze microglial activation, we resorted to standard immunohistochemical procedures. Activated microglia/macrophages were labeled using the antibody anti-rat CD68 (clone ED1, 1 : 500, Serotec, UK), which binds to an epitope on the lysosomal membrane of activated macrophages/microglia [38–40], rabbit anti-Iba1 (1 : 1000, WAKO), an antibody that recognizes a calcium binding protein present in the cytoplasm of microglia [41–43], and mouse anti-MHC-II (1 : 100, Serotec), an antibody that recognizes the major histocompatibility complex class II molecule [7].

Based on previous reports showing that pathological oligodendrocytes become Tau-1 positive after brain trauma and ischemia [44], we used the mouse anti-Tau-1 antibody (1 : 500, Chemicon, USA) to label dephosphorylated epitopes on damaged oligodendrocytes. Normal oligodendrocytes were labeled by mouse anti-Nogo-A (1 : 100, BD Transduction Lab, USA), an antibody that recognizes Nogo-A in the rat brain [45]. Myelin impairment was evaluated using an antibody against mouse anti-myelin basic protein (MBP), an important component of the compact myelin (1 : 100, Serotec, UK) [35].

Astrocytes were immunolabeled with an antibody rabbit antiglial fibrillary acid protein (GFAP, 1 : 1000, DAKO, UK), a classical astrocyte marker [18, 46, 47].

### 2.6. Immunolabeling Protocol

The slide-mounted sections were removed from the freezer, kept in an oven at 37°C for 30 minutes, and rinsed in 0.1 M phosphate buffer saline (PBS) for 5 min. To improve labeling intensity, sections were treated with 0.2 M boric acid (pH 9.0) previously heated to 65°C for 25 min. This temperature was maintained constant over the treatment period. Sections were allowed to cool down for about 20 min and incubated under constant agitation in 1% hydrogen peroxide solution in methanol for 20 min. Sections were then rinsed in 0.05% PBS/Tween (Sigma-Aldrich, USA) solution for 5 min for three times and incubated with 10% normal goat (GFAP, Iba-1) or horse (CD68, MBP, MHC-II, and Tau-1, Nogo-A) serum in PBS for 1 h.

Without further rinsing, sections were then incubated overnight with primary antibody in PBS, rinsed in PBS/Tween solution for 5 min (3 times), and incubated with biotinylated goat anti-rabbit (GFAP and Iba-1) or horse anti-mouse (ED1, MBP, MHC-II, Tau-1, Nogo-A) secondary antibodies (Vector Laboratories, USA) diluted at 1 : 200 or 1 : 100 in PBS, respectively, for 2 h. As a negative control, normal sera, rather the primary antibody, were used in some sections. Sections were rinsed again for 5 min (three times) and incubated in the avidin-biotin-peroxidase complex (ABC Kit, Vector Laboratories, USA) for 2 h. Sections were rinsed four times (5 min each) and revealed with DAB. After DAB reaction, sections were rinsed two times (5 min each) in 0.1 PB, dehydrated, and coverslipped with Entellan (Merck, Germany).

### 2.7. Qualitative Analysis

All sections stained with the different histological methods were surveyed by light microscopy (Nikon Eclipse E200). Illustrative images from all experimental groups were obtained using a digital camera attached to the microscope (Nikon Eclipse, 50i) using the software Moticam 2500®.

### 2.8. Quantitative Analysis

**Figure 1:**
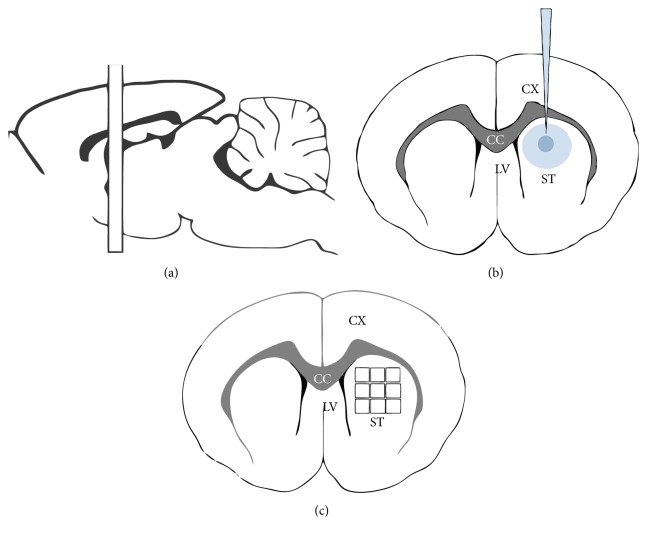
Schematic diagram illustrating the striatal injection site of ET-1 and the counting method. Drawing of a parasagittal section depicting the sectioning location (a), illustration of the ET-1 injection site in a coronal section drawing (b), and the location of the counting fields in the rat striatum (c). The squares in (c) are in scale to the counting fields they represent. CX = cortex; CC = corpus callosum; ST = striatum; LV = lateral ventricle.

We used coronal sections containing the damaged striatum located at 1.2 mm posterior to bregma to count the number of activated macrophages/microglia (CD68+ cells), MHC-II+ cells, pathological oligodendrocytes (Tau-1+ cells), Nogo-A+ cells, and astrocytes (GFAP+ cells) per field. Adjacent 20 *μ*m sections anterior and posterior to the lesion site were used for immunohistochemistry and cell bodies were counted over 1.5 mm from the lesion site. The counting field was defined as a square 0.25 mm wide grid (objective, ×40) in the eyepiece of a microscope. In the ×40 objective, this grid corresponds to an area of 0.0625 mm^2^. We counted 9 fields located at the dorsal striatum per section and 3 sections/animal (*N* = 5 animals per group) for all experimental groups (Figure 1).

For quantitative analysis of MBP immunolabeling, photomicrographs were segmented by Color Deconvolution plugin (Gabriel Landini, http://www.dentistry.bham.ac.uk/landinig/software/software.html) from ImageJ software version 1.33-1.34 (NIMH, NIH, Bethesda, MD, USA, https://imagej.nih.gov/ij/). After image segmentation, area values (*μ*m) and percentual fraction of DAB staining on sections were measured [48, 49]. We evaluated 5 sections/animal (*N* = 5 animals per group). Average values were expressed as mean ± S.E.M.

### 2.9. Statistical Analysis

Means and standard errors were calculated for all groups of data. Comparisons between different groups were assessed by analysis of variance (ANOVA) with Tukey* post hoc* test (one-way ANOVA-Tukey). Statistical significance was accepted at *p* < 0.05. All statistical analyses were performed using the Graphpad Prism 5.0 software.

## 3. Results

### 3.1. Focal Ischemia Induced by ET-1 Microinjections into the Rat Striatum

**Figure 2:**
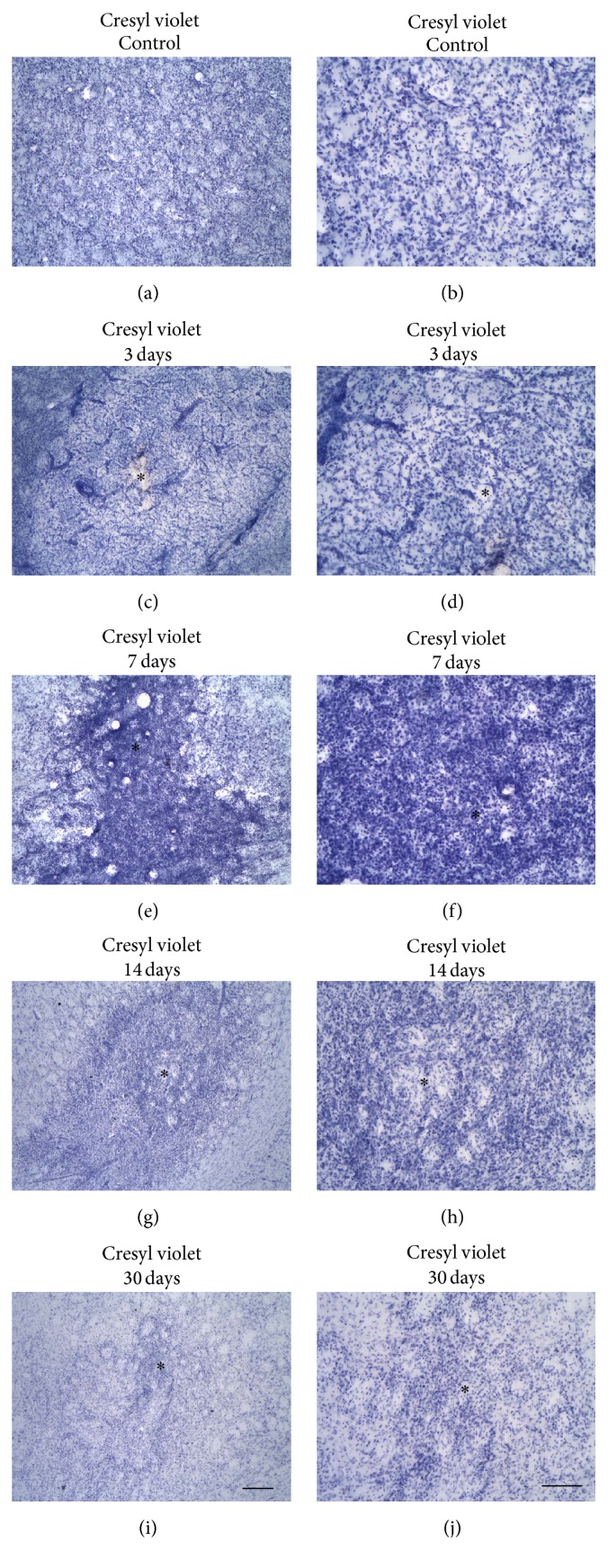
Gross histopathology after ET-1 microinjections revealed by cresyl violet staining. Control animal injected with sterile saline (a-b) or ischemic animals injected with ET-1 at 3 (c-d), 7 (e-f), 14 (g-h), and 30 (i-j) PLDs. Asterisks are in the lesion epicenter. Scale bars: (a), (c), (e), (g), and (i) (200 *μ*m) and (b), (d), (f), (h), and (j) (100 *μ*m).

ET-1 microinjections caused a focal ischemic damage characterized by tissue pallor, edema, intense inflammatory response, and loss of cell bodies (Figures 2(c)–2(j)). The tissue damage evolved from 1 day PLD with maximum infiltration of inflammatory cells between 3 and 7 PLDs (Figures 2(c)–2(f)). Mononuclear cells were present at 14 PLDs (Figures 2(g) and 2(h)), but there was a conspicuous decrease in their presence at 30 PLDs (Figures 2(i) and 2(j)). Control animals injected with sterile saline did not present these histopathological findings (Figures 2(a) and 2(b)).

### 3.2. ET-1 Microinjections Induce Progressive Astrocyte Activation in the Ischemic Striatum

**Figure 3:**
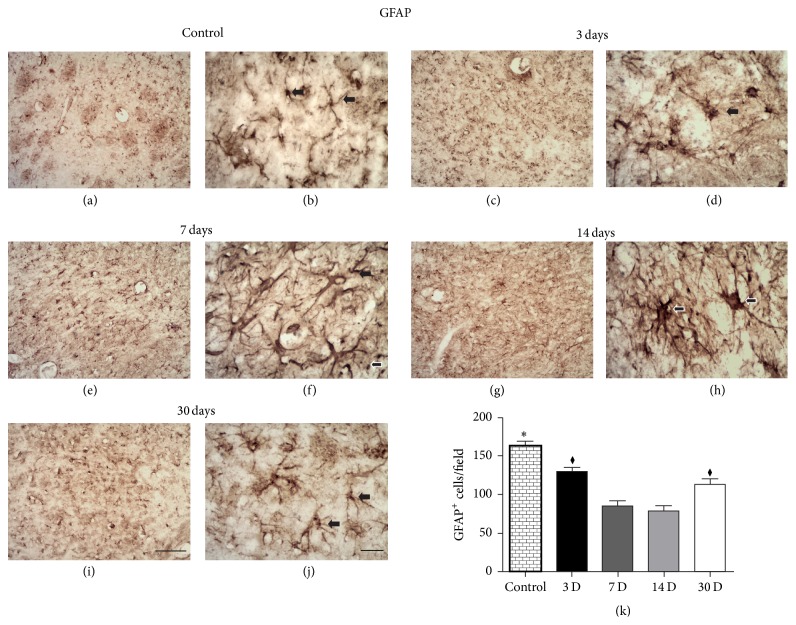
Progressive astrocyte activation after striatal focal ischemia. Control animal injected with sterile saline (a-b) or ischemic animals injected with ET-1 at 3 (c-d), 7 (e-f), 14 (g-h), and 30 (i-j) PLDs. Maximum astrocyte activation occurred around 7 and 14 PLDs. The cell density was smaller at 7 and 14 PLDs. ^*∗*^ *p* < 0.05 compared to others groups; ^*♦*^ *p* < 0.05 compared to 7 and 14 PLDs. Arrows point to astrocytes in the higher power images. Scale bars: (a), (c), (e), (g), and (i) (100 *μ*m) and (b), (d), (f), (h), and (j) (20 *μ*m).

In vehicle animals, astrocytes presented their normal morphology with nonhypertrophic cell bodies and a ramified pattern of their branches (Figures 3(a) and 3(b)). In ischemic animals, there was a progressive astrocyte activation starting from 3 PLDs (Figures 3(c) and 3(d)), with peak between 7 (Figures 3(e) and 3(f)) and 14 PLDs (Figures 3(g) and 3(h)) and clear reduction at 30 PLDs (Figures 3(i) and 3(j)). This activation was characterized by increased cell body volume and ramifications, mainly at 7 (Figures 3(e) and 3(f)) and 14 PLDs (Figures 3(g) and 3(h)).

One-way ANOVA revealed significant changes in the variance of the number of GFAP+ cells (*F*_4_ = 28.55; *p* < 0.0001). There was a decrease in the number of GFAP+ cells at 3 PLDs (129.2 ± 5.65) compared to vehicle animals (163.2 ± 6.13; *p* < 0.05). A further decrease occurred from 3 PLDs to 7 (85.00 ± 6.06; *p* < 0.001) and 14 PLDs (78.20 ± 6.93; *p* < 0.001) PLDs. There was a rise in the number of GFAP+ cells at 30 PLDs (113.2 ± 7.38) compared to 7 and 14 PLDs.

### 3.3. Microinjections of ET-1 Induce Microglia Activation up to 30 Days after Focal Ischemia

**Figure 4:**
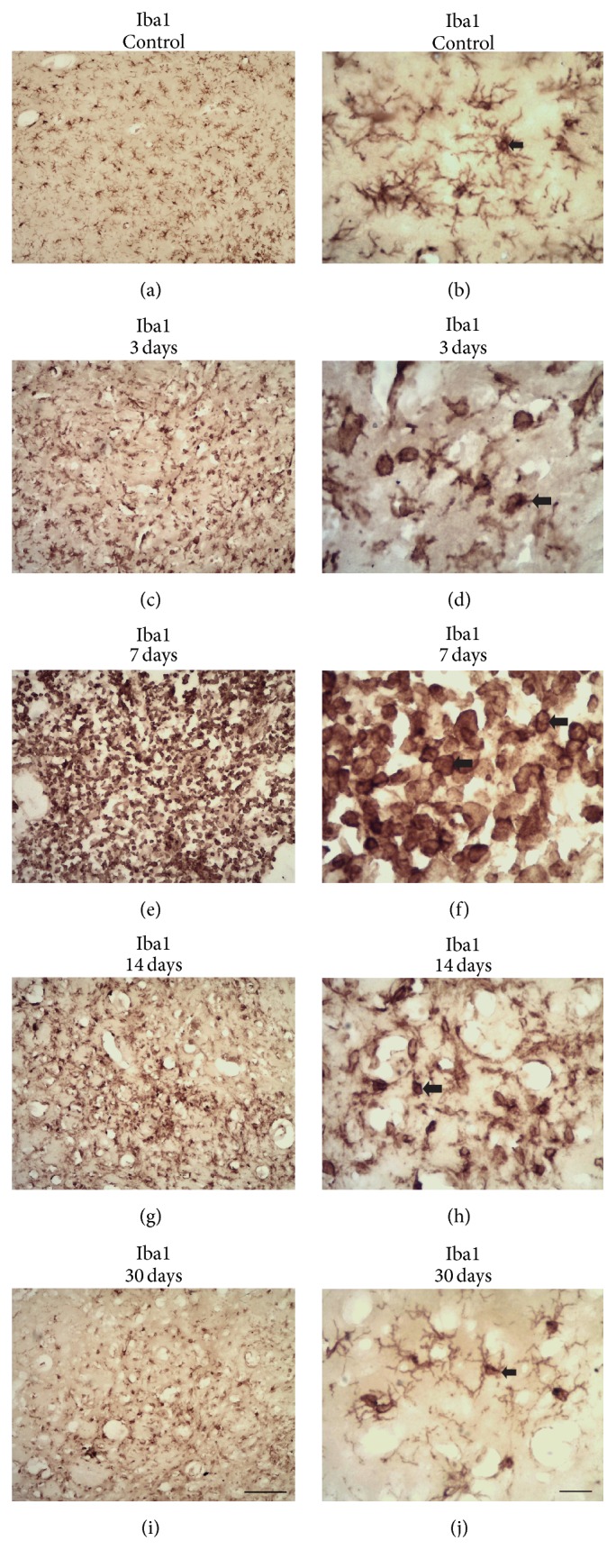
Progressive microglia activation after striatal focal ischemia revealed by Iba1 immunohistochemistry. Ramified microglia in a control animal injected with sterile saline (a-b). Morphological activation of microglia at 3 (c-d), 7 (e-f), 14 (g-h), and 30 (i-j) PLDs. Maximum activation occurred around 7 PLDs (e-f). Microglia activation decreased by 30 PLDs (i-j). Arrows point to Iba1+ ramified microglia (b, j) or round macrophages (d, f, h). Scale bars: (a), (c), (e), (g), and (i) (100 *μ*m) and (b), (d), (f), (h), and (j) (20 *μ*m).

Microglial activation was investigated using immunohistochemistry for 3 antibodies: Iba1, CD68, and MHC-II. In control animals, microglia displayed a ramified morphology with small Iba1+ cell bodies, which are characteristic features of resting microglia (Figures 4(a) and 4(b)). After focal ischemia, there was a progressive activation of microglia from 3 PLDs up to 14 PLDs (Figures 4(c)–4(h)). At 30 PLDs, microglia displayed morphological features of resting microglia (Figures 4(i) and 4(j)). The peak of microglia activation was around 7 PLDs (Figures 4(e) and 4(f)).

The Iba1 immunohistochemistry revealed the evolution of the morphological activation of microglia in ischemic animals. At 3 PLDs, there was a decrease in ramified Iba1+ cells in the ischemic site, with increase in amoeboid and round Iba1+ cells (Figures 4(c) and 4(d)). The amount of round Iba1+ cells (phagocytes) dramatically increased at the ischemic core at 7 days PLDs (Figures 4(e) and 4(f)). There was a progressive decrease in microglia activation at 14 (Figures 4(g) and 4(h)) and 30 PLDs (Figures 4(i) and 4(j)). At this later PLD, ramified Iba1+ cells were again observed in the ischemic site, despite the presence of some round and amoeboid microglia (Figures 4(i) and 4(j)).

**Figure 5:**
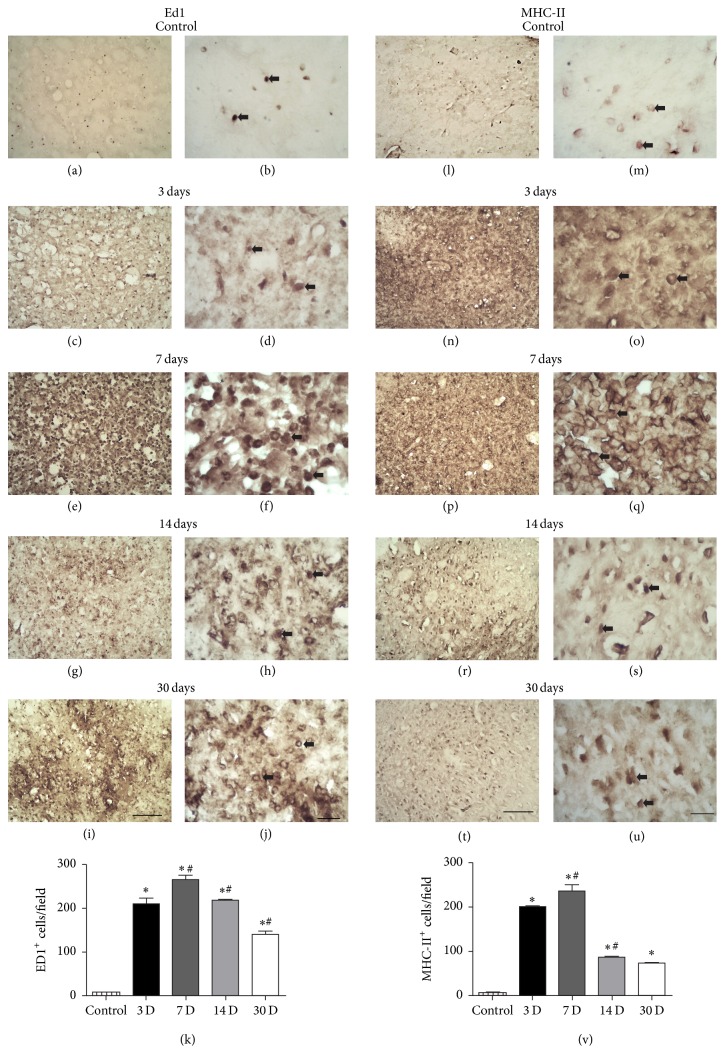
Microglia activation revealed by anti-CD68 and MHC-II immunohistochemistry. Control animals injected with sterile saline (a-b, l-m). Morphological activation of CD68+ or MHC-II+ microglia at 3 (c-d, n-o), 7 (e-f, p-q), 14 (g-h, r-s), and 30 (i-j, t-u) PLDs. Both techniques are labeled activated round macrophages (arrows). Quantitative analysis showed maximum number of cells at 7 PLDs with decrease at later survival times (k, v). ^*∗*^ *p* < 0.05 compared to control; ^#^ *p* < 0.05 compared to previous survival time. Scale bars: (a), (c), (e), (g), (i), (l), (n), (p), (r), and (t) (100 *μ*m) and (b), (d), (f), (h), (j), (m), (o), (q), (s), and (u) (20 *μ*m).

To quantitatively describe the temporal evolution of microglia activation following striatal ischemia, we counted the number of CD68+ cells between 3 and 30 PLDs (Figure 5(k)). The temporal immunoreactivity of CD68+ cells followed that described using the anti-Iba1 antibody (Figures 5(i) and 5(j)). One-way ANOVA revealed significant changes in the variance of the number of CD68+ cells (*F*_4_ = 96.35; *p* < 0.0001). The average number of CD68+ cells was 6.44 ± 0.29 cells/field in vehicle animals (Figures 5(a), 5(b), and 5(k)). Subsequent Tukey's* post hoc* comparisons indicated that this number increased to 209.70 ± 13.90, 266.40 ± 9.8, and 218.40 ± 1.69 cells/field at 3, 7, and 14 PLDs, respectively, (*p* < 0.001, Figure 5(k)). There was a considerable decrease in the number of CD68+ cells at 30 PLDs (140.70 ± 7.14; *p* < 0.001), but it remained elevated in comparison to control animals (6.44 ± 0.29; *p* < 0.001, Figures 5(i)–5(k)).

After ET-1 microinjections, there was a progressive expression of MHC-II in the ischemic striatum. The temporal profile of MHC-II expression was similar to those described with Iba1 and CD68, with maximum number of cells at 7 PLDs (Figures 5(l)–5(u) and 5(v)). MHC-II+ cells displayed morphology of activated round microglia (Figures 5(n)–5(u)). The average number of MHC-II+ cells was 5.77 ± 1.06 cells/field in control animals (Figure 5(v)).

One-way ANOVA revealed significant changes in the variance of the number of MHC-II+ cells (*F*_4_ = 153.1; *p* < 0.0001).* Post hoc* comparisons indicated that there was a progressive increase in the number of MHC-II+ cells per field at 3 and 7 PLDs, (201.26 ± 1.54; 236.57 ± 14.58; *p* < 0.001, Figure 5(v)). These numbers decreased at 14 and 30 PLDs, respectively (86.20 ± 2.69; 73.20 ± 1.98, *p* < 0.001, Figure 5(v)). All ischemic groups statistically differed from control animals. There was no statistical difference between 14 and 30 PLDs (*p* > 0.05, ANOVA-Tukey).

### 3.4. Microinjections of ET-1 Induced Progressive Impairment of MBP Immunoreactivity in the Ischemic Striatum

**Figure 6:**
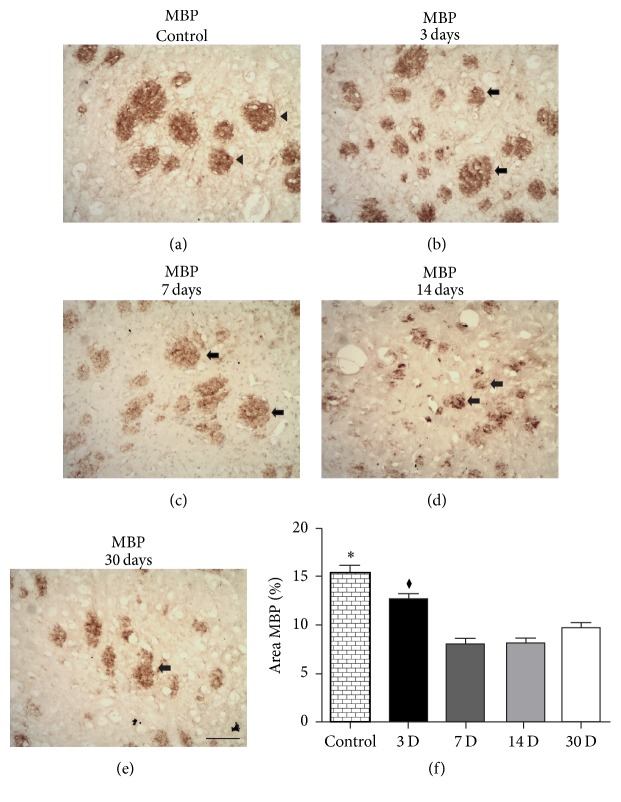
Myelin impairment revealed by anti-MBP immunohistochemistry. Control animal injected with sterile saline (a) or ischemic animals injected with ET-1 at 3 (b), 7 (c), 14 (d), and 30 (e) PLDs. There was a progressive impairment of MBP labeled WM tracts (arrows) compared to control (arrow in (a)). Quantitative analysis in (f). ^*∗*^ *p* < 0.05 compared to others groups; ^*♦*^ *p* < 0.05 compared to others survival time. Arrows point to WM tracts. Scale bars: 100 *μ*m.

The immunolabeling of the myelin sheath was performed using an anti-MBP antibody [35]. One-way ANOVA revealed significant changes in the variance of the values for MBP immunoreactivity in the striatum of ischemic animals (*F*_4_ = 28.97; *p* < 0.0001). Subsequent Tukey's* post hoc* comparisons indicated that there was a progressive impairment of MBP immunoreactivity after focal ischemia in the rat striatum from 3 days (12.67 ± 0.59; *p* < 0.05, Figure 6(b)) up to 30 days (9.71 ± 0.54; *p* < 0.001, Figure 6(e)) PLDs. The loss of MBP immunoreactivity was maximum at 7 (8.01 ± 0.57; *p* < 0.001, Figure 6(c)) and 14 PLDs (8.17 ± 0.46; *p* < 0.001, Figure 6(d)), but myelin impairment could be also observed at 30 PLDs (Figure 6(e)).

### 3.5. Microinjections of ET-1 Induce Oligodendrocytes Damage after Striatal Ischemia

**Figure 7:**
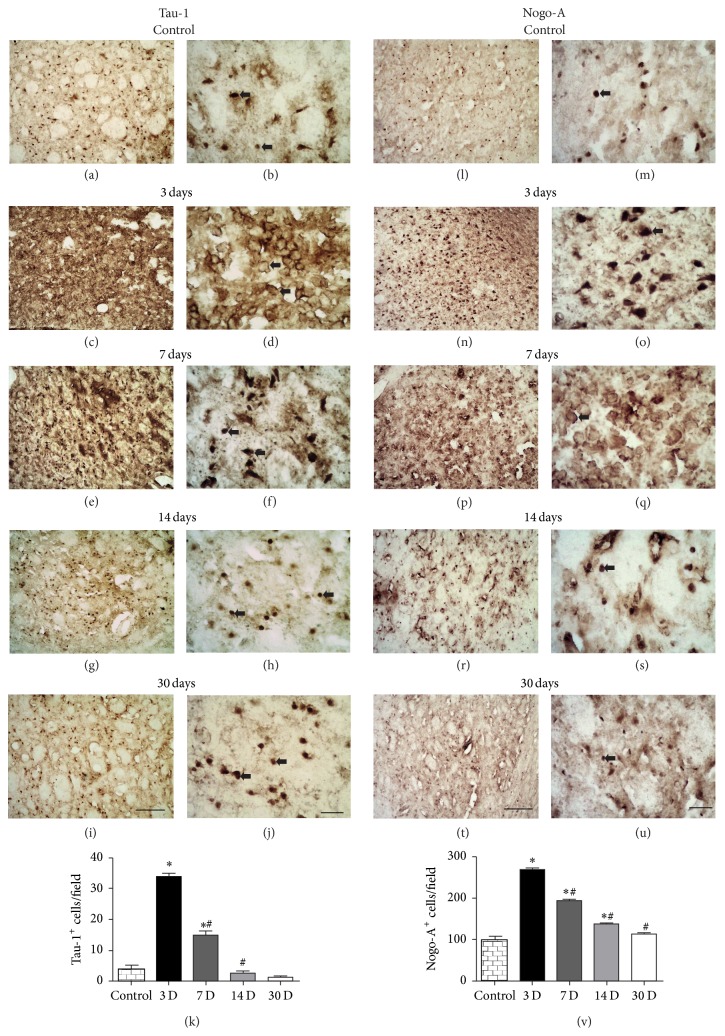
Oligodendrocyte damage (Tau-1+ cells) and increased Nogo-A immunoreactivity after focal striatal ischemia. Control animals injected with sterile saline (a-b, l-m). Tau-1 and Nogo-A+ cells at 3 (c-d, n-o), 7 (e-f, p-q), 14 (g-h, r-s), and 30 (i-j, t-u) PLDs. Quantitative analysis showed maximum numbers of Tau-1 and Nogo-A+ cells at 3 PLDs (k, v). There is a long-lasting increase in the number of Nogo-A+ cells up to 30 PLDs compared to control (*p* < 0.05, ANOVA-Tukey). ^*∗*^ *p* < 0.05 compared to control; ^#^ *p* < 0.05 compared to previous survival time. Scale bars: (a), (c), (e), (g), (i), (l), (n), (p), (r), and (t) (100 *μ*m) and (b), (d), (f), (h), (j), (m), (o), (q), (s), and (u) (20 *μ*m).

It has been shown that Tau-1 is a marker of pathological oligodendrocytes after acute damage to CNS [44, 50, 51]. Control animals expressed a small amount (3.773 ± 1.095/field) of Tau-1+ cells in the striatum (Figures 7(a), 7(b), and 7(k)). Ischemic damage induced a considerable increase in Tau-1 immunoreactivity in the acute phase after focal ischemia (Figure 7). One-way ANOVA revealed significant changes in the variance of the number of Tau-1+ cells in the striatum of ischemic animals (*F*_4_ = 153.1; *p* < 0.0001). Subsequent Tukey's* post hoc* comparisons indicated that the number of Tau-1+ cells/field was 33.56 ± 1.51 at 3 PLDs (*p* < 0.001, Figures 7(c), 7(d), and 7(k)). This number decreased to 14.80 ± 1.49, 2.59 ± 0.55, and 1.46 ± 0.22 at 7, 14, and 30 PLDs compared to 3 PLDs (*p* < 0.001, Figure 7(k)).

### 3.6. The ET-1-Induced Striatal Ischemic Damage Results in Progressive Increase in Nogo-A Immunoreactivity

Nogo-A is an oligodendrocyte-associated protein involved in the inhibition of axonal growth after acute CNS damage [31, 34]. One-way ANOVA revealed significant chances in the variance of the number of Nogo-A+ cells in the striatum of ischemic animals (*F*_4_ = 206.2; *p* < 0.0001). Subsequent Tukey's* post hoc* comparisons indicated that microinjections of ET-1 induced progressive increase in the number of Nogo-A+ cells with peak around 3 PLDs (267.59 ± 5.69; *p* < 0.001), with subsequent decrease at 7 (193.8 ± 3.980; *p* < 0.001), 14 (137.9 ± 137.9; *p* < 0.001), and 30 (112.8 ± 3.39; *p* < 0.001) PLDs (Figures 7(l)–7(u)). The average number of Nogo-A+ cells in control animals was (97.11 ± 10.47). All ischemic groups statistically differed from the control animals, even at 30 PLDs (Figure 7(v)).

## 4. Discussion

We have investigated the patterns of glial activation and Tau-1 and Nogo-A immunoreactivity from 3 up to 30 days after microinjections of ET-1 into the striatum of adult rats. The ET-1 model of focal ischemia uses the vasoconstrictor actions of the peptide ET-1 [52, 53]. This peptide causes focal ischemic damage after microinjections into the CNS [54], which is suitable for reproducing the focal strokes frequently occurring in the human brain [55]. In addition, the ET-1 model is easily performed without the complicated surgical procedures of other stroke models like the MCAO intraluminal filament technique, which may induce hyperthermia and other surgical complications [56]. This stroke model has been used in several publications by our group [3, 4, 12, 13].

We thought it would be important to establish the temporal evolution of microglia activation, astrocytosis, oligodendrocyte damage, and Nogo-A immunoreactivity during the first month after ET-1-induced stroke, as a morphological basis for further functional studies seeking new neuroprotective drugs.

Striatal ET-1 microinjections induced progressive astrocyte activation, as revealed by GFAP immunohistochemistry. Maximum activation was present in the first two weeks with a decline at 30 PLDs. This is in agreement with a previous study using the MCAO stroke model [57]. Nevertheless, in this study, the description of GFAP immunoreactivity was incomplete. No illustrations were provided for all survival times. Here, we filled this gap providing a comprehensive description of astrocyte activation from 3 to 30 days after experimental stroke onset.

The mechanisms by which astrocytes are activated after stroke and other neural disorders are not established. The meaning of this glial cell activation has been a matter of continuous debate for many years [58]. These cells are an important component of the glial scar in chronic survival times after CNS stroke and trauma [59]. The expression of proteoglycans is a biochemical barrier for axonal regeneration concomitant with a mechanical impediment imposed by intense astrocyte reaction and some degree of proliferation [8, 59]. The proliferation of these glial cells is influenced by factors including survivin [60]. Astrocytes may release deleterious molecules including the proinflammatory cytokine tumor necrosis factor-*α*, metalloproteinases, and reactive oxygen derived species [58, 61].

Nevertheless, astrocyte activation has also beneficial functions after stroke and other neural disorders [58, 62]. Astrocytes seem to contribute to neuroblast migration to striatum after stroke by releasing stroma-derived factor-1*α* (SDF-1) [63, 64] and monocyte chemoattractant protein 1 (MCP-1) [63]. This is important considering that partial replacement of neuronal loss is afforded by adult neurogenesis after striatal damage [65]. In addition, these glial cells can uptake glutamate in both physiological and ischemic conditions, buffering neurotoxicity [66]. Astrocyte may also release growth factors, including brain-derived neurotrophic factors (BDNF), contributing to neuron survival after stroke in rats [67].

There was intense microglia activation following microinjections of ET-1 into the rat striatum between 3 and 30 days after ischemic onset. Maximum activation occurred around 7 PLDs, but microglia remained activated up to 30 PLDs. These results are in agreement with previous studies using ET-1 stroke model [3, 4, 54] or MCAO [7, 68].

Microglia activation is a general response to any kind of CNS damage [11]. In nonpathological conditions, these brain resident macrophages patrol the CNS using stochastic movements of their fine branches [69, 70]. In pathological conditions like ischemia and trauma, microglia change their morphology becoming amoeboid and highly phagocytic round cells (macrophages) [11, 71]. Concomitant with morphological alterations, microglia change their genetic machinery expressing several types of receptors on their membranes, increased levels of transcription factors, and cytoplasmic enzymes [11].

We have labeled microglia using three antibodies: Iba1, ED1, and MHC-II. Iba1 immunohistochemistry allowed labeling the general population of microglial cells [41]. ED1 and MHC-II labeled a population of activated microglia [7, 39]. ED1 and MHC-II+ cells comprise both macrophages derived from the resident microglia and blood-borne macrophages [72]. These markers do not differentiate the origin of activate macrophages present in the ischemic striatum. It has been shown, using chimeric animals, that resident microglia first respond to the damage followed by hematogenous macrophages [72, 73].

The reasons by which microglia are both detrimental and beneficial after stroke are unknown. Recently, we have hypothesized these glial cells might be beneficial and/or detrimental in different pathological niches following striatal ischemia, a phenomenon likely determined by different concentrations of specific ligands along the pathological environment, which might act on different receptors in the microglia membrane [11]. This is in agreement with a recent study showing that M1 and M2 microglia phenotypes are present in the ischemic striatum following MCAO [74].

Microinjections of ET-1 into the rat striatum induced damage of oligodendrocytes (maximum at 3 PLD) and progressive myelin impairment (maximum between 7 and 14 PLDs). It seems that myelin impairment follows damage to oligodendrocyte cell bodies. It has been shown that oligodendrocytes present structural changes in their cytoskeleton (dephosphorylation) following ischemia, excitotoxicity, and trauma, which is visualized as an increased immunoreactivity for Tau-1 [44, 50, 75]. Tau-1 immunoreactivity has been frequently used as a specific marker of damaged oligodendrocytes [51, 54, 76]. We [51] and other authors have shown that this pathological phenomenon occurs in the acute phase after stroke [54, 76], which is in agreement with the present results. In addition, damage to axonal cylinder may precede both oligodendrocyte damage and myelin impairment. It has been shown that damage to axonal cylinder occurs in an early time point (6 hours) after focal striatal damage induced by ET-1 [72].

The increased immunoreactivity for Tau-1 in oligodendrocytes may be an early event underlying oligodendrocyte pathology [77]. It has been shown that oligodendrocytes but not astrocytes are labeled by Tau-1 after ischemia [77]. The increased immunoreactivity for Tau-1 in oligodendrocytes may represent an attempt to preserve oligodendrocyte cytoskeleton likely from free radicals-mediated damage [51]. This is in agreement with a previous report showing that Tau protein is fundamental for microtubule assembly [78] and that this structure is especially vulnerable to ischemia induced oxidative stress [75, 79]. The decrease in the number of Tau-1+ cells in later survival times in this study may be due to the loss of oligodendrocytes by apoptosis. This hypothesis is supported by a previous report of our group showing that striatal oligodendrocytes become apoptotic in later survival times following excitotoxic damage and that minocycline treatment decreases the number of Tau-1+ cells and partially reverses the apoptotic damage [51]. Several other authors have shown that oligodendrocytes undergo apoptosis following acute neural disorders [80–82]. Further studies should be performed to investigate the mechanisms by which oligodendrocytes are damaged after stroke and to address the therapeutic potential of neuroprotective drugs in reducing the WM demise.

The results show an increased number of Nogo-A+ cells in the acute phase after ET-1 induced stroke. This number remained elevated up to 30 days compared to nonischemic animals. This is in agreement with previous report using the MCAO filament model [45, 83]. The expression of Nogo-A in both neurons and oligodendrocytes is correlated with reduced neuroplasticity after SCI [25, 34, 84] and stroke [31, 83, 85]. Both early and delayed treatments of animals with monoclonal antibodies against Nogo-A increase neuroplasticity in these acute neural disorders [25, 31, 32, 34, 83–85]. Nevertheless, Nogo-A may also be involved in the survival of neurons after ischemia [33]. It follows that the increased expression of Nogo-A in the ischemic striatum can be related to both detrimental and beneficial actions. Further studies should consider this dual role of Nogo-A before the use of experimental approaches to neutralize the action of this protein.

## 5. Conclusion

Microinjections of ET-1 into the rat striatum induced conspicuous tissue loss, concomitant with progressive microglia and astrocyte activation, myelin impairment, oligodendrocyte damage, and Nogo-A up regulation. These pathological events were described from 3 to 30 days after ET-1-induced focal striatal ischemia. The establishment of the temporal evolution of these neuropathological events is an important step for future studies in which the manipulation of neuroinflammation and Nogo-A expression may be performed to enhance neuroprotection (in gray and white matter), adult neurogenesis, and functional recovery following striatal ischemic damage. We are addressing these issues in undergoing investigations.
